# Wide Anterior Maxillary Reconstruction with Equine Bone Xenograft: A Case Report of 24-Month Follow-Up

**DOI:** 10.1155/2020/8890935

**Published:** 2020-10-21

**Authors:** Davide De Cicco, Giuseppe Colella, Gianpaolo Tartaro, Nicola Zerbinati, Romolo Fragola, Raffaele Rauso

**Affiliations:** ^1^Maxillofacial Surgery, University of Naples “Federico II”, Naples, Italy; ^2^Maxillofacial Surgery Unit, University of Campania “Luigi Vanvitelli”, Naples, Italy; ^3^Dermatology, University of Insubria, Varese, Italy

## Abstract

*Introduction*. Orofacial reconstruction plays an important role in the treatment of patients affected by oral and maxillofacial cancers. Improvements in technologies and studies of biomaterials have widely expanded surgical possibilities to achieve good functional and aesthetic outcomes. By the way, xenografting procedures gained great consensus in the last decades, because of their documented reliability and efficacy. We present a case of anterior maxillary chondrosarcoma (CHS) that has undergone surgical ablation followed by reconstruction with an equine-derived bone xenograft. *Case Presentation*. A 68-year-old woman affected by CHS of the premaxilla underwent surgical ablation involving the four incisors followed by reconstruction using an equine-derived bone substitute. Bony reconstruction was planned to achieve implant and dental prosthetic rehabilitation at a second surgical time. Primary surgery was carried out without complications. Good integration of the graft was confirmed by radiological examination. At 12-month follow-up, the patient refused the implant placement and spontaneously adopted a mobile prosthesis. One year later, plates and screws were removed, because of the exposure of a titanium plate. The graft was finally rejected within 3 weeks. *Discussion*. Nonantigenic equine-derived biomaterials have shown reliability and a good safety profile. In the presented case, implant insertion should have been performed 12 months after the primary surgery. During the follow-up, until dental mobile prosthesis was applied, clinical and instrumental examinations demonstrated a good integration of the graft. We suppose that a chronic inflammation of the mucosa led to the exposure of the plate, perhaps due to pressure, minimal movements, or imperfect fitting of the mobile prosthesis. Removal of fixation means was performed to prevent grafting failure, without success. On the other hand, missing load could induce the graft to act just like a prosthesis, without a real process of integration. Safety and reliability of equine-derived bone xenografts cannot be currently confirmed if not followed by implant insertion and dental rehabilitation.

## 1. Introduction

Nowadays, achieving satisfactory orofacial reconstruction has become mandatory to guarantee an acceptable quality of life for patients presenting oral and maxillofacial cancers, especially in cases were the alveolar process of the maxilla, and of course the teeth, are involved in surgical ablation [1]. Historically, prosthetic solutions were commonly used to restore maxillary defect after ablative surgery, until free tissue transfer has become the gold standard in hard and soft tissue reconstruction due to the “like with like” criterion [2]. In those subjects not suitable for free flaps or autologous grafts, adequate functional and aesthetic outcomes can be achieved choosing among several heterologous options which gained large consensus over the last years, thanks to lower donor site morbidity and reduction of surgical time [3–5].

Improvement in technologies during the last decades widely expanded possibilities in surgical reconstruction with a number of different bone grafting procedures and biomaterials, such as xenogenic bone substitutes [6]. The current literature shows the useful role of xenografting procedures in bone regeneration and reconstruction, because of its osteoconductive properties, serving like a scaffold to guide new bone formation [7–11]. Advantages in using xenogenic materials are represented by donor site morbidity and operation time reduction [6]. However, long-term follow-up evaluations are poorly documented in the literature [12], probably due to the scarce popularity, if compared to autologous grafting procedures. Although their efficacy and reliability have been already discussed, researches are still ongoing.

We present a case of upper maxillary chondrosarcoma (CHS) resection, reconstructed by using equine-derived bone xenograft, with a 24-month follow-up.

## 2. Case Presentation

**Figure 1:**
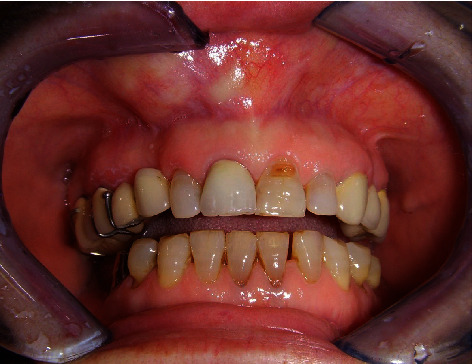
Clinical preoperative assessment. Swelling of the maxillary alveolar process is clearly shown just above the central and lateral incisors.

**Figure 2:**
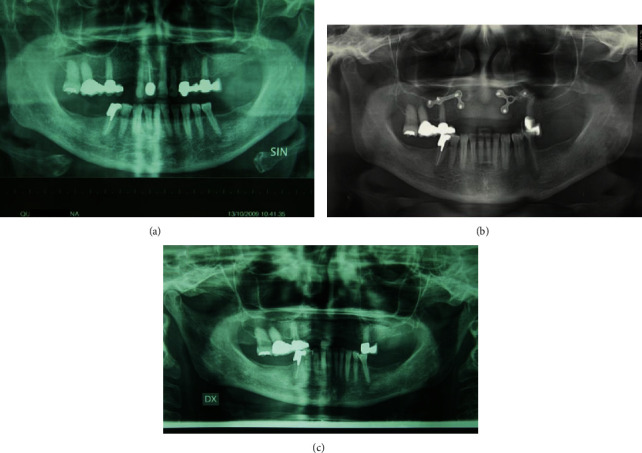
(a) Preoperative panoramic X-ray documenting an osteolytic lesion of the premaxilla. (b) Postoperative panoramic X-ray documenting the correct position of graft and fixation means. (c) Panoramic X-ray after removal of plates and screws (24-month follow-up) revealing signs of resorption and lower radiopacity of the graft material.

**Figure 3:**
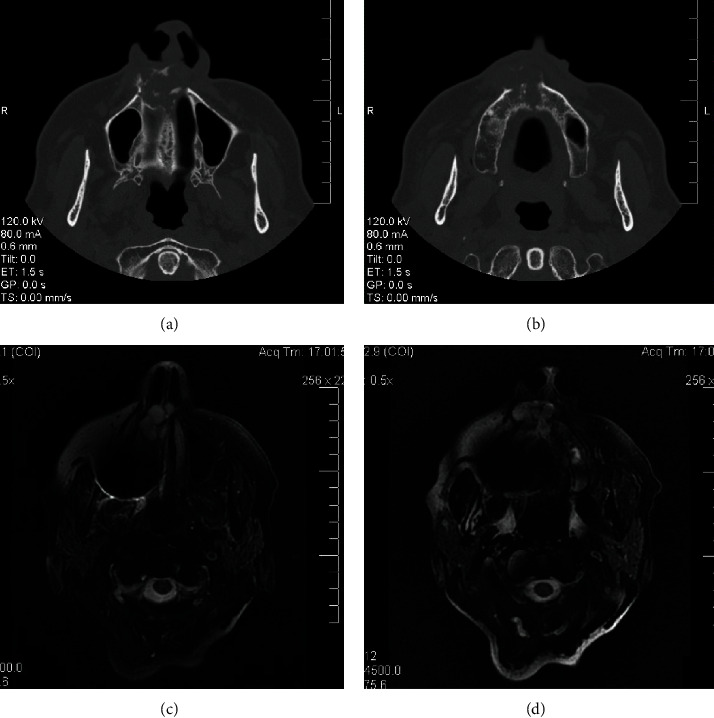
(a, b) Preoperative CT scan. (c, d) Preoperative MRI.

**Figure 4:**
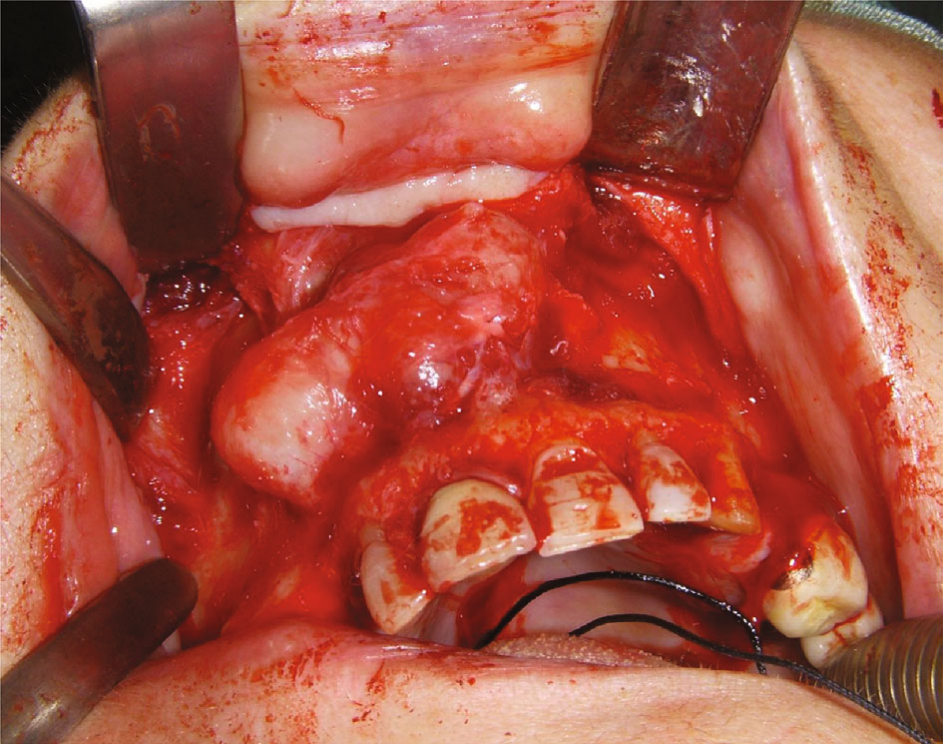
Intraoperative appearance of the neoplasm after the elevation of the mucoperiosteal flap, carried out until the exposure of the pyriform aperture.

**Figure 5:**
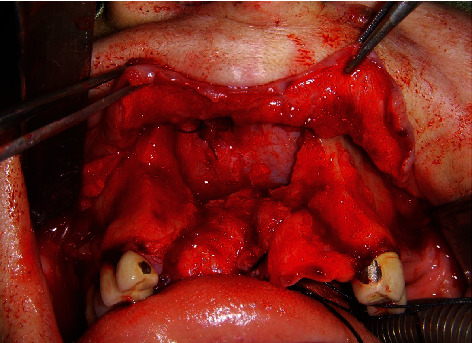
Surgical defect after resection of the neoplasm, involving the anterior nasal floor.

**Figure 6:**
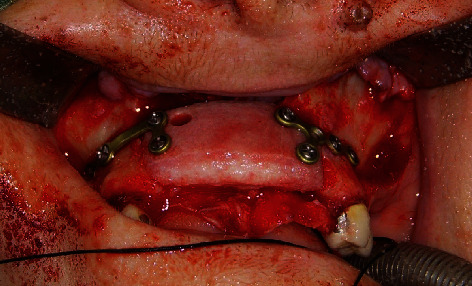
Equine xenograft has been fixed by one titanium plate and four screws per side.

**Figure 7:**
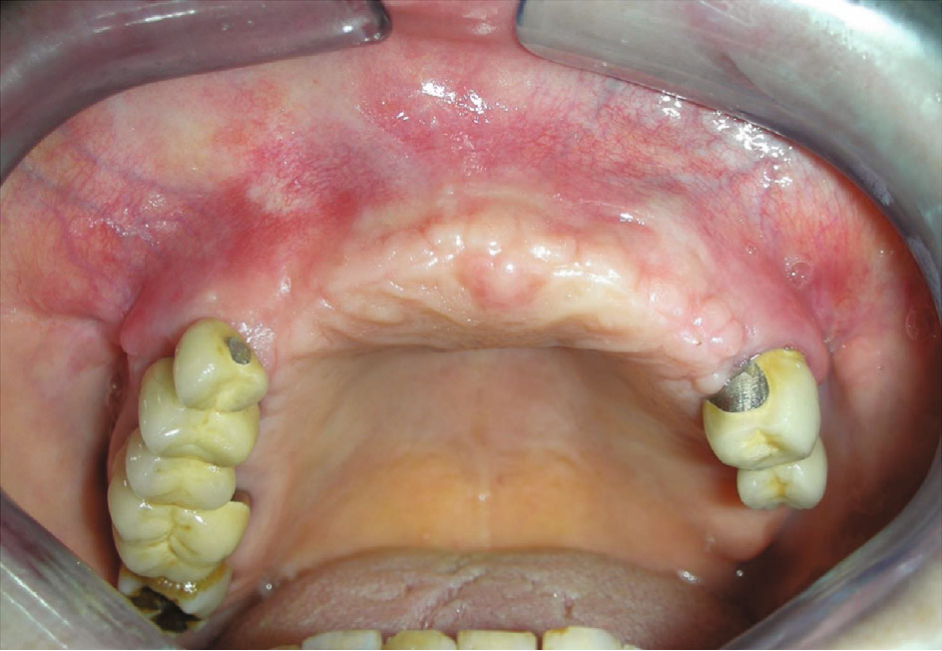
Postoperative complete wound healing observed 2 weeks after surgery.

**Figure 8:**
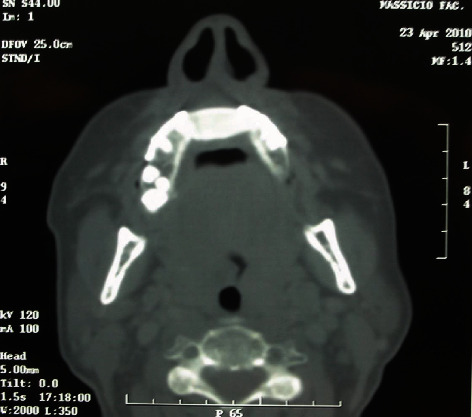
Postoperative CT scan, acquired 5 months after surgery, showing intense radiopacity and good integration of the grafted material.

A 68-year-old woman with controlled hypertension, was referred in November 2009 at the Maxillofacial Surgery Unit of the Second University of Naples (currently renamed University of Campania “Luigi Vanvitelli”). Clinically, the patient presented a midfacial swelling, especially in the upper lip and nasal areas. Intraoral inspection revealed swelling of the anterior alveolar process of the maxilla between the two lateral incisors (Figure 1), extended vertically until the anterior nasal floor. Panoramic X-ray (Figure 2(a)), CT scan (Figures 3(a) and 3(b)), and MRI (Figures 3(c) and 3(d)) documented an osteolytic lesion of the anterior alveolar process of the maxilla, 2.5 cm in width and 3.5 cm in height, extended cranially on the anterior maxilla and the anterior nasal floor. Surgical procedure was performed in December 2009 in general anesthesia, and complete removal of the neoformation was achieved (Figures 4 and 5). Surgical defect measured 3.5 cm in width and 4 cm in height. It was restored by using equine-derived cortical and spongy bone graft with preserved type 1 collagenic component (OSTEOPLANT® Bioteck S.p.A., Arcugnano, Italy) prepared starting from an equine femoral head cut by using Pirzosurgery® (Mectron, Vicenza, Italy), which has recently demonstrated better results among cutting devices [13]. After implantation, the graft was fixed bilaterally by using titanium plates and screws (Figure 6). No soft tissue defects were present. Complete wound healing was obtained without complications (Figure 7), and postoperative panoramic X-ray (performed 1 week later) showed good fixation and stability of the graft (Figure 2(b)). CT scan performed 5 months after surgery confirmed the adequate integration of the graft (Figure 8).

**Figure 9:**
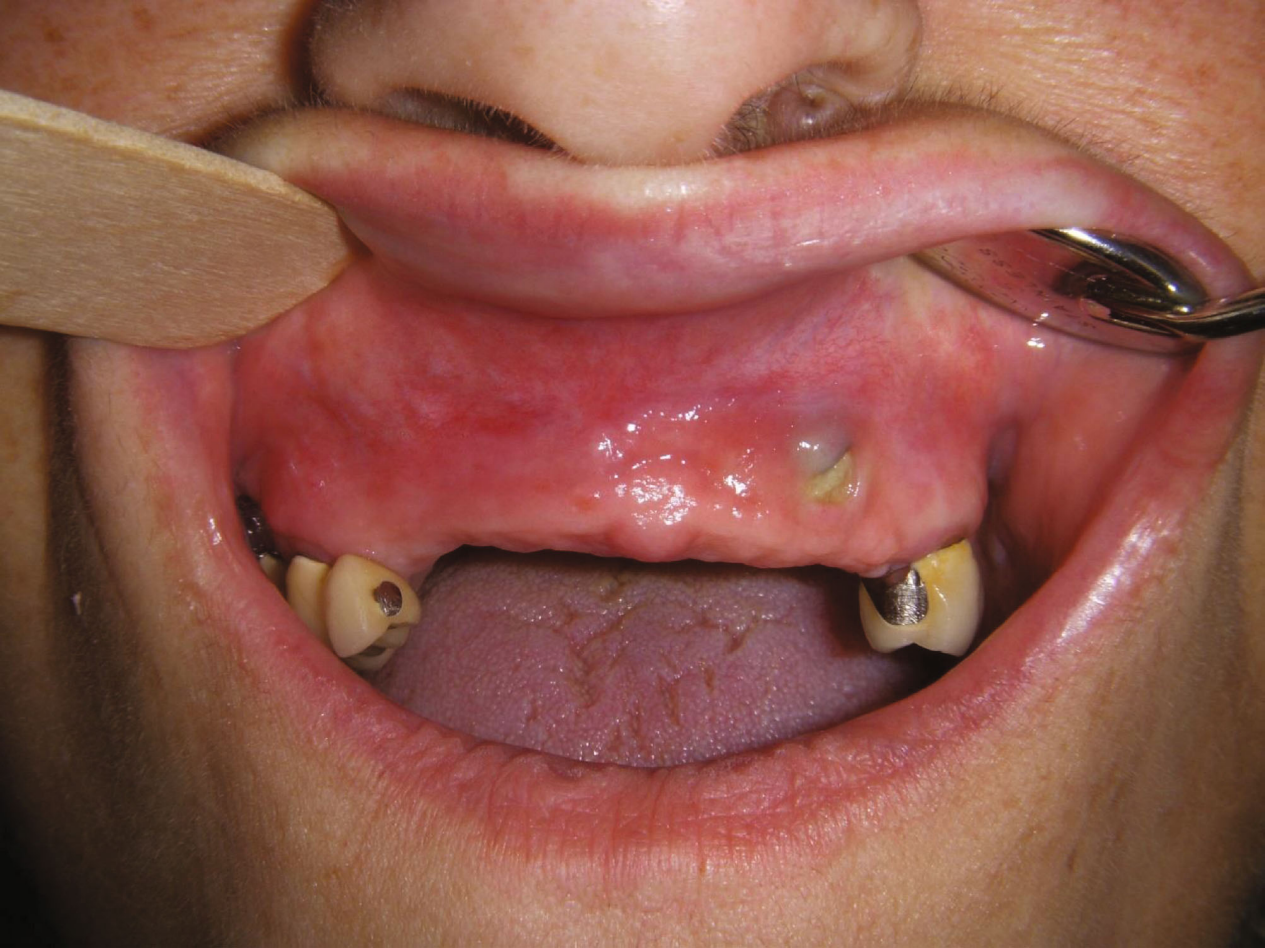
Exposure of the left-placed plate occurred 24 months after surgery (18 months after applying the mobile prosthesis).

**Figure 10:**
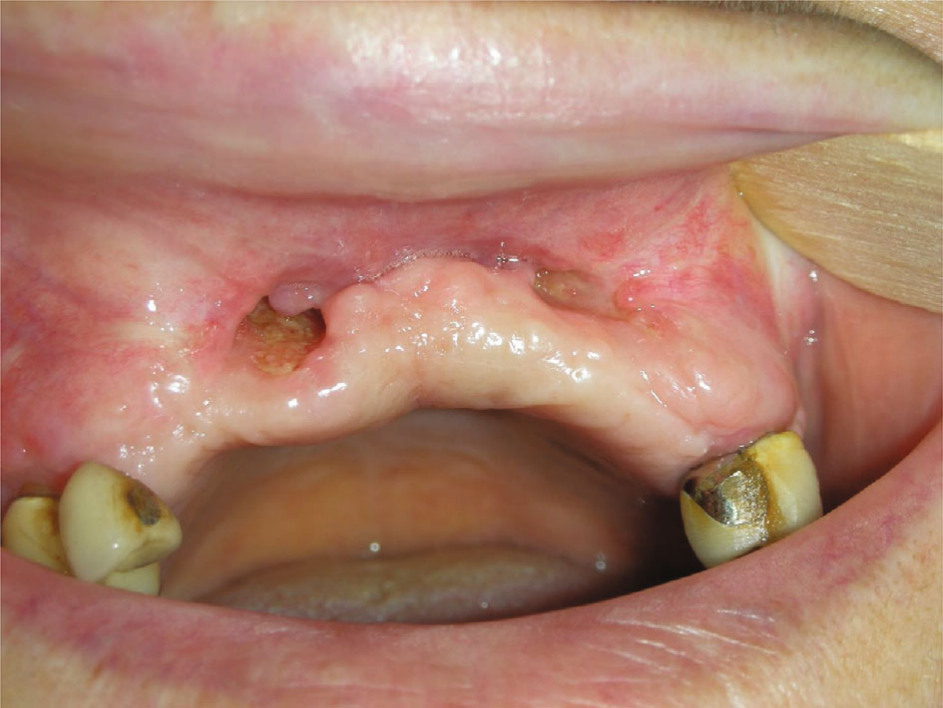
Postoperative dehiscence occurred after the removal of plates and screws, widely exposing the underlying equine graft.

**Figure 11:**
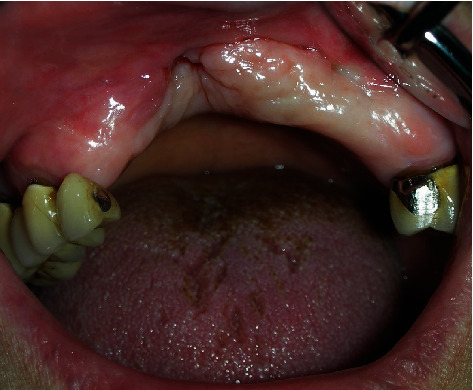
Intraoral appearance 2 weeks after graft rejection. Anterior mucosal envelope collapsed due to the lack of structural support, showing the wide anterior maxillary defect.

One year after surgery, dental implant placement was planned preoperatively, although the patient refused it and spontaneously adopted a mobile prosthesis 6 months after surgery. At 24-month follow-up, 20 months after mobile prosthesis application, exposition of the left titanium plate became clinically evident, as well as first signs of graft resorption revealed by the X-ray evaluation (Figures 2(c) and 9). Plates and screws were removed in order to prevent complete graft failure, but this intervention was complicated by an ample mucosal dehiscence and graft was rejected within 3 weeks from surgery (Figures 10 and 11).

## 3. Discussion

Chondrosarcoma (CHS) is a primary bony malignant tumor characterized by neoplastic cells producing hyaline cartilage [14, 15]. Among all cases of CHS described in the literature, only 1% are referred to the jaws [16]; thus, it is considered a rare and poorly documented lesion [17]. This slow-growing lesion presents high local invasiveness and mild clinical presentation that often lead to a tardive diagnosis with huge tumors already at the first medical observation. The most common symptoms are swelling of the involved area, pain, and facial asymmetry [17]. Prognosis of affected patients is defined by histological type (six different patterns have been described), tumor grade, recurrence, presence of metastasis (even if really rare), and quality of the surgical treatment [17]. Regarding treatment solutions, wide surgical resection of the tumor is considered the gold standard, since it is associated with the best prognosis [17].

The gold standard in maxillary reconstruction following surgical ablation is represented by composite bony free flaps for defects exceeding 5-6 cm in size or by autologous or heterologous bone grafts for smaller ones [18].

The ideal reconstruction should closely reproduce the biological characteristics of human bone. Therefore, autologous grafting is widely considered the gold standard for the reconstruction of posttraumatic and postoncological bone defects, also being the only solution combining together osteogenic, osteoconductive, and osteoinductive properties [19]. However, the current literature showed high variability in respect of grade, timing, and occurrence of graft resorption [20]; thus, autografts are considered unpredictable by most. Moreover, they require long surgical time and have an intrinsic risk of donor site morbidity, teeth injuries, and iatrogenic fractures [21]. In the presented case, this kind of reconstruction has been proposed, but the patient refused due to psychological factors.

Historically, the development of alternative materials has represented a field of great interest. Nowadays, surgeons can choose among several bony substitutes: allografts, xenografts, or synthetic materials. All these options based their principle of function on their osteoconductive properties [6]. Indeed, bony substitutes are acellular materials acting like scaffolds for osteocyte migration and production of new formed bone.

Most studies described the use of bone substitutes in implant dentistry for alveolar ridge augmentation and not for restoration or large maxillary oncological defects. Thus, the presented case could be better compared with other craniofacial reconstructive procedures, such as neurosurgical cranioplasty that provides several examples of grafting procedure using bone substitutes [22]. However, to the best of our knowledge, equine-derived bone grafts have not been documented in published literature and could be considered for further clinical trials, as it has demonstrated optimal outcomes even in orthopedics [23].

Allografts showed adequate results in implant dentistry as well as in other fields [20, 24, 25]. The major drawback of this option is the risk of immunological reactions given by the protein component. Many authors advocated the risk of disease transmission, although it has not been reported in published literature [26]. Allografts are obtained from human cadavers or donors and stored in allogenic bone banks. In the presented case, the unavailability of allogenic bone substitutes forced us to evaluate other solutions.

The synthetic materials arouse great interest, because of their reliable and reproducible chemical composition and microscopic architecture [27].

PMMA is a biocompatible and resorbable substance that allows new bone formation. However, as the substitute is resorbed, the newly formed bone is susceptible to fractures and is considered most suitable for restoration of small defects [20]. Some studies advocated that bacterial contamination could be responsible for biofilm formation leading to a high risk of infections [19, 20]. The possibility of covering this material with an antibiotic coat during the production process seems to be able to overcome the issue [19].

The most studied materials for bone reconstruction are calcium phosphate ceramics, as well as hydroxyapatite (HA) and *β*-tricalcium phosphate (*β*-TCP) [27]. HA is widely considered a useful solution in secondary cranioplasty, providing a stable osteoconductive scaffold for bone regeneration, being resistant to resorption [26]. *β*-TCP also bases its principle of function on osteoconductive properties. Additionally, some studies reported an osteoinductive potential and conversely to HA, it is quickly resorbed by human osteoclasts with subsequent new bone formation [27].

Equine-derived bone substitutes have shown optimal results in oral surgery and a good safety profile [7, 8, 11, 28–32]. Unlike almost all other heterologous bone grafts, they have also demonstrated osteoinductive properties and the preservation of several growth factors which enhance osteocyte colonization and new bone development [33]. We experienced satisfactory outcomes in the reconstruction of the upper and lower jaws using these bone substitutes; thus, it has been chosen among the others, even though consensus on the preferred material is lacking in the current literature [20].

The graft used in the presented case is developed from equine femoral head deantigenated throughout a multistep enzymatic process (Zymo-Teck®, Bioteck S.p.A., Arcugnano, Italy) that allows a selective removal of all potential antigenic components of the row material ensuring a full biocompatibility. Sequential treatments in solutions containing glycolytic and lipolytic enzymes are conducted in pressurized washing cycles at low controlled temperature. The product is then processed through an oxidative phase by using hydrogen peroxide that allows the removal of cellular debris and contaminants. In the final step, grafts are freeze-dried, placed in a double blister pack, and sent to *β*-ray sterilization.

Pistilli et al. [32] reported a case of severe bilateral maxillary atrophy treated by equine-derived bone grafting followed up by clinical and radiological evaluations over a period of 14 months. Implants were placed 8 months after primary surgery, and prosthetic load was achieved in 3 months. Fourteen months after primary surgery, radiological examination documented a complete preservation of the bone graft. Furthermore, the authors performed a biopsy of the grafted material during the second surgical step. Histological examination revealed the presence of vital newly formed bone, no inflammatory tissues, and active remodeling of the grafted material showing the ongoing integration, in parallel with others' findings [28, 29].

In the presented case, a secondary implant insertion should have been performed 12 months after the primary surgery, but the patient refused implants due to psychological distress following the first operation.

During the follow-up, until a dental mobile prosthesis was applied, clinical and instrumental examinations (CT scan and radiographs) revealed good healing and biointegration of the graft. After applying a mobile prosthesis for 12 months (at 24-month follow-up), left plate exposure was observed. We suppose that it could be related to the pressure and minimal movements of the overlying dental prosthesis once applied; this could have caused a chronic inflammation of the mucosa inducing the underlying plate to be exposed. However, unilateral development of this delayed complication suggests that the mobile prosthesis could have fitted imperfectly discharging excessive pressure on the left titanium plate. Removal of plates and screws before the application of the mobile prosthesis could have prevented the exposure and subsequent grafting failure. On the other hand, it could be interesting to note that, although a total integration of the graft is advocated, in the presented case, 24 months after the xenograft placement, it still was not completely substituted by native bone tissue.

Moreover, it is mandatory to consider that bone xenografts have been introduced as a surgical alternative of autologous bone graft, but they have been studied almost exclusively in implant dentistry, so it could be assumed that lack of bone loading could have profound consequences on xenograft integration [34]. Missing load could induce the graft to act just like a prosthesis, without a real process of integration and autologous bone replacement.

It would be interesting to compare the presented case with others; however, to the best of our knowledge, no cases of xenogenic bone grafting without subsequent dental implant rehabilitation have been reported in the literature.

From the present study, although it is limited to a case report, some interesting issues arise: the safety and reliability of equine bone xenograft (do not used for further implants insertion) cannot be confirmed currently; and in cases of bone grafting, both autologous or heterologous, it could be reasonable to remove plates and/or wires used to fix the graft before dental mobile prosthesis application.

## Additional Points

*Human and Animal Rights*. All research procedures followed were in accordance with the Helsinki Declaration of 1964.

## References

[1] Moreno M. A., Skoracki R. J., Hanna E. Y., Hanasono M. M. (2009). Microvascular free flap reconstruction versus palatal obturation for maxillectomy defects. *Head & Neck*.

[2] Granick M. S., Husovski J. (2012). *Wound Healing for Plastic Surgeons, An Issue of Clinics in Plastic Surgery, Volume 39-3*.

[3] Brown J. S., Shaw R. J. (2010). Reconstruction of the maxilla and midface: introducing a new classification. *The Lancet Oncology*.

[4] Vincent A., Burkes J., Williams F., Ducic Y. (2019). Free flap reconstruction of the maxilla. *Seminars in Plastic Surgery*.

[5] Pogrel M. A., Podlesh S., Anthony J. P., Alexander J. (1997). A comparison of vascularized and nonvascularized bone grafts for reconstruction of mandibular continuity defects. *Journal of Oral and Maxillofacial Surgery*.

[6] Dimitriou R., Jones E., McGonagle D., Giannoudis P. V. (2011). Bone regeneration: current concepts and future directions. *BMC Medicine*.

[7] de Azambuja Carvalho P. H., dos Santos Trento G., Moura L. B., Cunha G., Gabrielli M. A. C., Pereira-Filho V. A. (2019). Horizontal ridge augmentation using xenogenous bone graft—systematic review. *Oral and Maxillofacial Surgery*.

[8] Esposito M., Grusovin M. G., Coulthard P., Worthington H. V. (2006). The efficacy of various bone augmentation procedures for dental implants: a Cochrane systematic review of randomized controlled clinical trials. *The International Journal of Oral & Maxillofacial Implants*.

[9] Esposito M., Grusovin M. G., Felice P., Karatzopoulos G., Worthington H. V., Coulthard P. (2009). The efficacy of horizontal and vertical bone augmentation procedures for dental implants—a Cochrane systematic review. *European Journal of Oral Implantology*.

[10] Lima R. G., Lima T. G., Francischone C. E., Turssi C., Assis N., Sotto-Maior B. (2018). Bone volume dynamics and implant placement torque in horizontal bone defects reconstructed with autologous or xenogeneic block bone: a randomized, controlled, split-mouth, prospective clinical trial. *The International Journal of Oral & Maxillofacial Implants*.

[11] Giudice R. L., Rizzo G., Centofanti A. (2018). Steam sterilization of equine bone block: morphological and collagen analysis. *BioMed Research International*.

[12] Naenni N., Lim H. C., Papageorgiou S. N., Hämmerle C. H. F. (2019). Efficacy of lateral bone augmentation prior to implant placement: a systematic review and meta-analysis. *Journal of Clinical Periodontology*.

[13] Lo Giudice R., Puleio F., Rizzo D. (2019). Comparative investigation of cutting devices on bone blocks: an SEM morphological analysis. *Applied Sciences*.

[14] Chow W. A. (2018). Chondrosarcoma: biology, genetics and epigenetics. *F1000Research*.

[15] Gadwal S. R., Fanburg-Smith J. C., Gannon F. H., Thompson L. D. R. (2000). Primary chondrosarcoma of the head and neck in pediatric patients: a clinicopathologic study of 14 cases with a review of the literature. *Cancer*.

[16] Kumar M., Suresh K., Patil M., Pramod R., Yusuf R., Bilahari N. (2014). Mesenchymal chondrosarcoma of posterior maxilla: report of a case with brief literature review. *Annals of Medical and Health Sciences Research*.

[17] de Souza L. L., Pontes F. S. C., Fonseca F. P., da Mata Rezende D. S., Vasconcelos V. C. S., Pontes H. A. R. (2019). Chondrosarcoma of the jaw bones: a review of 224 cases reported to date and an analysis of prognostic factors. *International Journal of Oral and Maxillofacial Surgery*.

[18] Foster R. D., Anthony J. P., Sharma A., Pogrel M. A. (1999). Vascularized bone flaps versus nonvascularized bone grafts for mandibular reconstruction: an outcome analysis of primary bony union and endosseous implant success. *Head & Neck*.

[19] Lobb D. C., DeGeorge B. R., Chhabra A. B. (2019). Bone graft substitutes: current concepts and future expectations. *The Journal of Hand Surgery*.

[20] van de Vijfeijken S. E. C. M., Münker T. J. A. G., Spijker R. (2018). Autologous bone is inferior to alloplastic cranioplasties: safety of autograft and allograft materials for cranioplasties, a systematic review. *World Neurosurgery*.

[21] Kloss F. R., Offermanns V., Kloss-Brandstätter A. (2018). Comparison of allogeneic and autogenous bone grafts for augmentation of alveolar ridge defects—a 12-month retrospective radiographic evaluation. *Clinical Oral Implants Research*.

[22] Ganau M., Cebula H., Fricia M. (2020). Surgical preference regarding different materials for custom-made allograft cranioplasty in patients with calvarial defects: results from an internal audit covering the last 20 years. *Journal of Clinical Neuroscience*.

[23] Santini S., Barbera P., Modena M., Schiavon R., Bonato M. (2011). Equine-derived bone substitutes in orthopedics and traumatology: authors’ experience. *Minerva Chirurgica*.

[24] Shibuya N., Jupiter D. C. (2015). Bone graft substitute: allograft and xenograft. *Clinics in Podiatric Medicine and Surgery*.

[25] Cockerham B., Patel A., Greenwell H., Hill M., Shumway B., Hsu H. (2020). Ridge augmentation comparing a cancellous block allograft to an osteoinductive demineralized bone matrix allograft: a randomized, controlled, blinded clinical trial. *The International Journal of Periodontics & Restorative Dentistry*.

[26] Sanz M., Vignoletti F. (2015). Key aspects on the use of bone substitutes for bone regeneration of edentulous ridges. *Dental Materials*.

[27] Bohner M., Santoni B. L. G., Döbelin N. (2020). *β*-Tricalcium phosphate for bone substitution: synthesis and properties. *Acta biomaterialia*.

[28] Rothamel D., Schwarz F., Herten M. (2009). Vertical ridge augmentation using xenogenous bone blocks: a histomorphometric study in dogs. *The International Journal of Oral & Maxillofacial Implants*.

[29] Zecha P. J., Schortinghuis J., van der Wal J. E. (2011). Applicability of equine hydroxyapatite collagen (eHAC) bone blocks for lateral augmentation of the alveolar crest. A histological and histomorphometric analysis in rats. *International Journal of Oral and Maxillofacial Surgery*.

[30] Cortese A., Pantaleo G., Borri A., Caggiano M., Amato M. (2016). Platelet-rich fibrin (PRF) in implant dentistry in combination with new bone regenerative technique in elderly patients. *International Journal of Surgery Case Reports*.

[31] Di Stefano D. A., Artese L., Iezzi G. (2009). Alveolar ridge regeneration with equine spongy bone: a clinical, histological, and immunohistochemical case series. *Clinical Implant Dentistry and Related Research*.

[32] Pistilli R., Signorini L., Pisacane A., Lizio G., Felice P. (2013). Case of severe bone atrophy of the posterior maxilla rehabilitated with blocks of equine origin bone: histological results. *Implant Dentistry*.

[33] Pacaccio D. J., Stern S. F. (2005). Demineralized bone matrix: basic science and clinical applications. *Clinics in Podiatric Medicine and Surgery*.

[34] Robling A. G., Turner C. H. (2009). Mechanical signaling for bone modeling and remodeling. *Critical Reviews in Eukaryotic Gene Expression*.

